# Recommendations on Sexuality and Intimacy After Burn Injuries

**DOI:** 10.3390/ebj7020026

**Published:** 2026-05-12

**Authors:** Jill Meirte, Stefania Anna Simone, Sabrina Belemkasser, Jonathan Bayuo

**Affiliations:** 1 OSCARE—Organisation for Burns, Scar Aftercare & Research, 2170 Antwerp, Belgium; 2 Department of Rehabilitation Sciences and Physiotherapy REVAKI-MOVANT, Faculty of Medicine and Health Sciences, University of Antwerp, 2610 Antwerp, Belgium; 3 Division of Plastic, Aesthetic and Reconstructive Surgery, University Hospital Graz, 8036 Graz, Austria; stefaniaanna.simone@uniklinikum.kages.at; 4 Burn Treatment Center, Saint-Louis Hospital, 75010 Paris, France; 5 School of Nursing and Midwifery, University of Southern Queensland, Ipswich 4305, Australia

**Keywords:** burn injury, sexuality, intimacy, body image, psychosocial rehabilitation, quality of life, patient communication, healthcare professional education, burn-care

## Abstract

**Highlights:**

**What are the main findings?**

- Sexuality, intimacy, and body image remain under-addressed but significantly affected domains in burn rehabilitation.
- An interdisciplinary, evidence-informed process resulted in practical communication tools validated with international multicultural stakeholder feedback.

**What are the implications of the main findings?**

- Burn rehabilitation should integrate proactive, culturally sensitive discussions on sexuality, intimacy, and body image.
- Practical tools and targeted training can support clinicians in addressing sexuality within patient-centered burn care.

**Abstract:**

Background: Burn injuries profoundly impact the survivors’ physical, psychological and social well-being, with sexuality, intimacy and body image remaining among the most disrupted yet least addressed areas of rehabilitation. Limited professional training, social discomfort, and a lack of clinical guidance contribute to these unmet needs. Recognizing sexuality as an essential part of health, this interdisciplinary project developed evidence-informed recommendations and communication tools to support both burn survivors and healthcare professionals in discussing intimacy, sexuality, and body image after burn injury. Methods: An interdisciplinary expert group conducted a narrative literature review, supplemented with grey literature and lived experience resources. These insights informed two practical tools: a poster for healthcare professionals and burn centers, and a patient-focused brochure. Both underwent iterative refinement through multicultural feedback from patients and professionals across Europe, Asia and Africa, followed by final validation during an interdisciplinary workshop at the 2025 European Burns Association (EBA) Conference. Results: The literature indicated that burn injuries affect sexuality and body image through interacting physiological, psychological, sensory, relational, and sociocultural factors. Common challenges included reduced desire, anxiety, fear of rejection, altered self-perception, and discomfort initiating conversations about intimacy. Professionals reported limited training, insufficient privacy and cultural barriers. Conclusions: The developed tools and these recommendations aim to normalize dialog, support proactive screening, and promote culturally sensitive patient-centered burn rehabilitation. Embedding sexuality and intimacy within burn care requires ongoing professional training and the identification of dedicated resource persons within each team.

## 1. Introduction

Burns are a unique type of injury at the intersection of dermatology and disability. Depending on the severity, patients may undergo major surgeries (multiple skin grafts, amputations, etc.) and have noticeable scars. These impairments induce extreme bodily changes and scars that individuals sometimes hide in everyday life. Thus, burn injury represents a life-changing event involving physical, behavioral, and social changes, as well as alterations in body image and sexuality [1]. Although advancements in intensive care, surgery, and rehabilitation have improved survival rates [2], burn survivors continue to face substantial long-term challenges. These include coping with changes to their appearance, physical functioning, social interactions, and personal identity. Among these challenges, sexuality remains one of the most affected and yet most frequently overlooked areas in burn care [3].

There is a real sense of social unease when this topic is raised, affecting not only patients but also healthcare professionals [4]. This leads to an avoidance of dialog with patients and a consequent lack of care in this area. Patients may feel ashamed and constantly hide their scars, which prevents them from speaking up about their relationship with their own body and thus their intimacy. This can lead to silent, intense suffering and impair their daily quality of life [5]. Yet, sexuality is an inherent part of human life and represents a central aspect of existence (WHO Sexuality), and the skin is a sensory and erotic organ that enables people to connect with themselves and the world. Given this, it is crucial to incorporate the field of sexuality, a significant health concern, in the comprehensive care of patients suffering from burns. We aim to contribute to this by drawing up specific recommendations, drawing on our multi-disciplinary viewpoints.

In our process, we will consider the concept of sexuality at a complex level, including sex, gender identities and roles, sexual orientation, eroticism, pleasure, intimacy and reproduction. Sexuality is experienced and expressed in thoughts, fantasies, desires, beliefs, attitudes, values, behaviors, practices, roles and relationships. While sexuality can include all of these dimensions, not all of them are always experienced or expressed. Sexuality is influenced by the interaction of biological, psychological, social, economic, political, cultural, legal, historical, religious and spiritual factors [6]. It seemed essential to us to insert concepts peripheral to sexuality, which would enable us to understand the patient’s experience in a less direct and potentially brutal way. With this in mind, the concept of intimacy is often a softer door through which to approach the question of sexuality with the patient. As far as professionals are concerned, intimacy covers a broader field that is easier to assess. Intimacy -or the state of being intimate in a personal and or private way- refers to the need and ability to experience and enjoy emotional closeness with others and have closeness also returned to you [7]. Moreover, given the major and brutal disruption of body reference points caused by burn injuries and its impact on the thorny question of the mirror, it is crucial to consider the concept of body image. Body image is the mental image an individual has of their own body, influenced by the observed/perceived social stereotypes [8]. It is also defined as the systematic, cognitive, affective, unconscious representation that people have concerning their bodies during their biological development and throughout their social relationships [9]. Body Image (BI) is a multidimensional concept that involves people’s positive and negative perceptions, thoughts, behaviors, and attitudes about their body and appearance [10]. At last, quality of life which is an individual’s perception of their position in life in the context of the culture and value systems in which they live, and in relation to their goals, expectations, standards, and concerns (WHOQOL group), enables us to link sexual issues to other essential aspects of daily life, and build bridges between different areas of a patient’s suffering.

Guided by these fundamental concepts, this recommendation seeks to address a critical gap in burn rehabilitation by offering clear, interdisciplinary guidance on incorporating sexuality, intimacy, and body image into holistic patient care. Our recommendation also aims at raising awareness among healthcare professionals about the importance of these frequently overlooked dimensions and to provide practical, culturally sensitive resources that facilitate meaningful conversations between professionals and patients.

## 2. Materials and Methods

### 2.1. Expert Group and Interdisciplinary Approach

The theme for this recommendation was first defined within the Professionals Allied to Medicine (PAM) Committee of the European Burns Association (EBA). When the PAM Committee was asked to revise the European Practice Guidelines for Burn Care (EBA, 2017), it became evident that topics related to sexuality and intimacy had not been addressed, highlighting a critical gap in existing burn care recommendations. The theme for this recommendation was first defined within the PAM Committee, following growing awareness among its members based on shared clinical experiences and international feedback that sexuality and intimacy, while critical aspects of recovery, were consistently being overlooked in burn rehabilitation. This recognition was grounded in the observation that, across clinical settings, professionals lacked clear protocols, designated personnel to address sexual health, and structured educational training on the topic. In addition, burn centers often lack culturally sensitive resources and accessible patient materials, leaving survivors without guidance or a protected space to discuss their intimate lives. In response, a multi-method, interdisciplinary approach was adopted to explore these unmet needs and to develop practical, evidence-informed solutions.

At first, an expert group was assembled, composed of four professionals with extensive experience in burn care and related fields, including an advanced practice nurse specializing in sexual medicine and therapy, a nursing science researcher, a physiotherapy assistant professor, and a clinical psychologist. All members had direct experience working with individuals recovering from burn injuries.

### 2.2. Literature Review

An extensive narrative literature review was conducted to investigate the complex and interrelated issues of sexuality and intimacy following burn injuries. The review aimed to synthesize current knowledge, identify key thematic trends, and highlight gaps in both the literature and clinical practice relevant to burn survivors and professionals.

Systematic research was performed in the electronic database PubMed in July 2024. To ensure the inclusion of recent studies, database alerts were established and monitored for new publications until May 2025, when the recommendation was finalized.

The search strategy employed Boolean operators (AND, OR, NOT) to combine keywords, including burn injuries, body image, sexuality, Intimacy, psychological impact. Reference lists of relevant articles were manually screened to identify additional studies.

The search focused on English-language publications. Grey literature and publicly available resources, such as those from the Sunshine (Social Welfare Foundation) Organization, the Model Systems Knowledge Translation Center (MSKT), and the Phoenix Society for Burns Survivors, were reviewed.

Based on preliminary findings and expert opinion, we defined the aim and scope of the project, which included raising awareness of the unmet needs of burn survivors regarding sexuality and Intimacy and providing practical, culturally sensitive resources for patients and healthcare professionals.

### 2.3. Tool Development and Validation Process

The expert group collaboratively aimed to develop evidence-informed recommendations and practical support tools.

These tools were created based on insights gained from the narrative literature review and the expert group’s clinical expertise and were further refined through feedback sessions with both patients and healthcare professionals. From the onset, the primary objective was to create resources that were meaningful, practical, and user-friendly for both audiences.

Draft versions were reviewed in multiple feedback sessions involving individuals at various stages of recovery across Europe, Asia, and Africa. To ensure accessibility and cultural sensitivity, the materials were translated into local languages and tested with users in their native contexts. Feedback was then integrated into the design, and materials were retranslated into English for further refinement. Only after the final adjustment was complete, the infographics were translated back into the local languages for broader dissemination. As this study focused on tool development and qualitative validation through expert and user feedback, no demographic or clinical variables (e.g., age, sex, TBSA, burn depth, length of hospitalization, or complications) were collected.

The feedback collected during this process was systematically discussed by the expert group, resulting in several iterative rounds of revision. This development approach emphasized usability, clarity, emotional resonance, and cultural appropriateness, ensuring that the final tools aligned closely with the lived experiences of patients and the communication needs of healthcare professionals.

The final versions of the tools were presented and validated during a face-to-face interdisciplinary workshop and round-table discussion at the European Burns Association (EBA) Conference in Berlin in September 2025. The session included clinicians working in various burn (after-care) centers, researchers, and patient advocates, with contributions in confirming the tools’ relevance, usability, acceptability and pointing out missing information. The tools were then finalized based on this collective input, marking the conclusion of the development and validation process.

**Figure 1:**
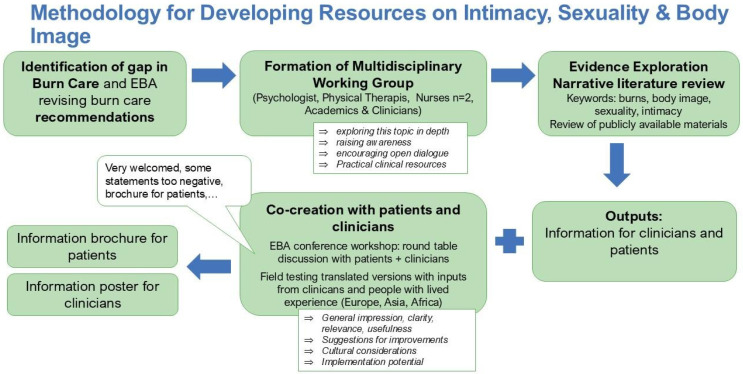
Methodology for Developing Resources on Intimacy, Sexuality and Body Image.

An illustration of the methodology for developing our resources can be found in Figure 1.

## 3. Results

### 3.1. Impact of Burns on Body Image

Burn survivors frequently experience feelings of shame, insecurity, and a desire to hide their scars due to altered body image. These feelings significantly contribute to their overall quality of life and psychosocial adjustment [5]. Major burn injuries often lead to significant changes in appearance, causing body image dissatisfaction (BID), which is a prominent issue among adult burn survivors. BID is linked to distress, negative perception of one’s body characteristics, and subsequent psychological and physical challenges [11]. There is a notable gender difference in body image dissatisfaction, with females reporting lower scores in body image domains and being particularly at risk. Burn severity, particularly in visible areas like the lower limbs and face, greatly impacts body image and is correlated with long-term dissatisfaction [12].

There is a causal relationship between body image and sexuality changes post-burn [12]. TBSA, burn depth, location of the burn or burn sites and post-burn scarring are all important clinical factors for topics such as body image/intimacy/sexuality [9]. Length of stay in the burn unit is also a factor to take into account. The longer the length of stay the more problems with body image dissatisfaction [13].

Burn injuries do not always result in body dissatisfaction, indicating the plasticity of body image and the ability to adjust in the burn population [14,15]. Burn survivors often experience a strong correlation between poor body image and lower sexual satisfaction. Body image dissatisfaction significantly affects the romantic and sexual aspects of their lives [16]. Altered body image plays a critical role in affecting sexual role functioning and satisfaction. However, some burn survivors may experience post-traumatic growth and adjust to their altered body image [9].

### 3.2. Impact of Burns on Intimacy and Sexuality

Burn injuries can have a profound impact on sexual function, encompassing a range of psychological, physiological, and sensory aspects. Following burn injury survival, common sexual and intimacy issues that may emerge include lack of desire for intimacy, low energy or sexual drive, diminished interest, fear of sexual performance, anxiety, and fear of rejection- often rooted in body image disturbances. When the burn injury involves the genitals, such as penile burns, pain and contractures can interfere with normal functioning [17,18,19]. Hordern [20] emphasizes the importance of a patient-centered approach to addressing sexuality, particularly in the context of life-altering or life-limiting conditions. She argues that sexuality should be recognized as a core component of holistic care, and that open, respectful communication between healthcare providers and patients is essential. Patients may feel isolated or ashamed of their sexual concerns, and without proactive engagement from clinicians, these issues often remain unaddressed. Hordern [20] also highlights that acknowledging the emotional and relational dimensions of sexuality—beyond physical function—is crucial for supporting patients in reclaiming a sense of identity and intimacy after trauma.

#### 3.2.1. Psychopathological Factors

Anxiety, depression, and post-traumatic stress disorder (PTSD) are frequently reported among burn survivors and are known to significantly impair sexual function. These conditions can lead to heightened stress, fear, and emotional distress, which interfere with sexual desire and performance [12,21]. Burn survivors often experience social withdrawal and reduced self-confidence, which further exacerbate difficulties in forming or maintaining intimate relationships [9].

#### 3.2.2. Psychodynamic Factors

Burn injuries often result in profound changes in body image and self-perception. Survivors may experience a sense of dehumanization and altered bodily boundaries, leading to psychodynamic regression. This can manifest as a rejection of sexual thoughts or activities and a diminished capacity for autonomous decision-making in intimate contexts. The damaged body becomes the object of multiple treatments, overwhelmed by numerous medical devices and thus dissociated from its emotional and erotic dimensions. It will take a long time and many steps for the subject to reclaim their body and recreate the link between their physiological body and their erogenous body and, thus, recreate a state of libidinal subversion [22], which is conducive to any sexual investment by the subject. Prior to this possibility, and for a long time, the sexual dimension is unthinkable for the patient, who remains, out of a need for survival, in a physiological dimension.

#### 3.2.3. Physiological Factors

Burn injuries can disrupt physiological processes essential for sexual function through multiple mechanisms. Severe burns trigger a strong and long-lasting stress response in the body. This stress reaction can interfere with normal hormone balance and reduce the production of sex hormones, resulting in reduced libido, fatigue, and decreased sexual responsiveness [23]. In addition, extensive burns can lead to alterations in endocrine regulation, metabolic imbalance, and vascular dysfunction, all of which may interfere with sexual arousal and performance.

Pharmacological treatments commonly used during burn recovery, including analgesics, antihypertensives, antidepressants, and anxiolytics, may further negatively influence sexual desire, arousal, and sexual function through central and peripheral neurochemical effects [19,24]. In male survivors with major burns, erectile dysfunction has been specifically noted in male patients with major burns, underscoring the need for long-term follow-up and multi-disciplinary management [24].

#### 3.2.4. Surgical Factors

Surgical interventions, particularly those involving the genital area or surrounding tissues, can result in anatomical changes, scarring, and contractures that physically hinder sexual activity. These complications may lead to pain during intercourse and reduced sexual satisfaction [19]. Moreover, reconstructive surgeries may not fully restore function or appearance, contributing to ongoing distress and avoidance of intimacy [1,12].

#### 3.2.5. Sensory Factors

Burn injuries often alter skin sensation, resulting in conditions such as dysesthesia (painful sensations), hypoesthesia (reduced sensation), and hyperesthesia (increased sensitivity). These sensory changes can make physical intimacy uncomfortable or even painful, further discouraging sexual activity [19]. Sensory impairments are particularly distressing when they affect erogenous zones or areas associated with self-image and attractiveness.

#### 3.2.6. Relational and Communication Barriers

Burn injuries can profoundly affect intimate relationships and partner dynamics. Survivors and their partners may struggle with changes in roles during recovery, fear of causing pain or emotional distress, altered patterns of physical closeness, and uncertainty about when and how to resume sexual activity. Concerns about scars, altered appearance, and perceived loss of desirability may lead to avoidance of intimacy and fear of rejection, even within previously stable relationships [16].

Communication barriers frequently arise both within relationships and in interactions with healthcare professionals. Many patients report reluctance to raise sexual concerns due to embarrassment, shame, or the belief that sexuality is no longer a legitimate concern following severe injury. At the same time, healthcare professionals may avoid the topic due to lack of training, discomfort, assumptions about patient priorities, or time and role constraints [18,25]. As a result, sexual health concerns often remain unaddressed unless explicitly and sensitively initiated by clinicians.

**Figure 2:**
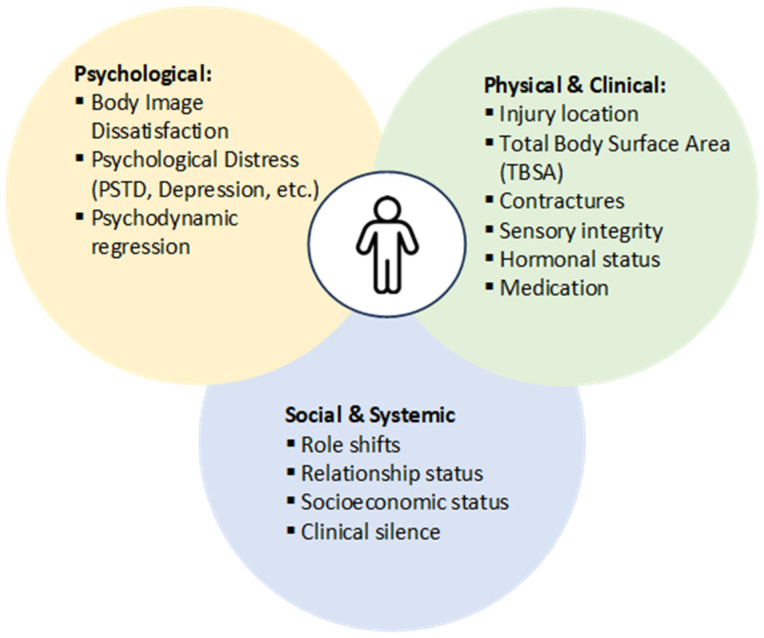
Multifactorial Determinants Affecting Sexuality and Intimacy Following Burn Injury.

The different factors affecting sexuality and intimacy following burn injuries are illustrated in Figure 2.

### 3.3. Predictive Factors of Sexual Dysfunction Following Burn Injuries

Sociodemographic variables such as age, gender, marital status, and employment status are important factors influencing intimacy, sexual function, and satisfaction in burn survivors. However, the literature presents inconsistent findings regarding the impact of age at the time of injury, with studies reporting both positive and negative associations [16]. Gender differences are more consistently reported. Female patients appear to experience a greater impact on sexual function. Both men and women report reduced sexual arousal at 6 and 12 months post-burn. However, men tend to experience a loss of sexual interest and discomfort with physical intimacy (e.g., hugging, hand-holding, kissing) during the early stages of rehabilitation, whereas women report similar issues predominantly at 12 months post-injury. Among severely burned individuals, women frequently report lower levels of sexual satisfaction, often associated with altered appearance and body image. In contrast, men tend to report better outcomes in domains such as sexual relationships, social interaction, employment, and romantic intimacy [26].

Furthermore, a higher proportion of men remain in sexual relationships following burn injuries [15]. Öster et al. [21] identified significant gender differences in the Burn Specific Health Scale–Brief (BSHS-B) sexuality subscale at 6 and 24 months post-burn, with women reporting lower satisfaction and men showing greater improvement over time. Men with genital burns also exhibit long-term urinary and sexual dysfunctions [27]. Marital status also plays a role. Single or divorced individuals of both sexes report lower sexual satisfaction. Being in a relationship at the time of injury is associated with higher sexual satisfaction scores at 12 and 24 months post-burn [28]. Employment status is another relevant factor; individuals who are unemployed are less likely to be sexually active and are less frequently in relationships [15].

Clinical factors such as total body surface area (TBSA) burned, length of hospital stay (LOS), scar location, and body image perception are also predictive of sexual dysfunction. A TBSA greater than 20% is strongly associated with sexual dysfunction. The visibility and location of scars significantly affect psychological adjustment [29]. Hidden scars may lead to anxiety about discovery, particularly in children [19,30]. Burns affecting the genitalia or other visible areas are linked to sexual dysfunction [29]. In women, burns on the chest and breast area are often perceived as the most disfiguring [19].

Hospitalization duration also correlates with sexual satisfaction. Ahmad et al. [31] found statistically significant associations between LOS and sexual satisfaction, with dissatisfaction increasing in patients hospitalized for more than 60 days. More severe burns requiring prolonged hospitalization are associated with lower scores on the BSHS-B sexuality subscale, with adverse effects persisting up to seven years post-injury [21,29]. Finally, satisfaction with appearance and body image is strongly linked to sexual satisfaction [32]. Psychiatric comorbidities and coping strategies also significantly influence sexual outcomes [21].

### 3.4. Perception of Burn Care Staff on Intimacy and Sexuality

Issues related to intimacy and sexuality are generally neglected by healthcare professionals in the care of patients [3]. A scoping review by Bayuo et al. [16] highlights the limited body of scientific research addressing this topic in burn care settings, identifying only 13 studies focused on burn survivors or patients with burn injuries, and just 4 studies that examined the perspectives of burn care staff. Notably, there is a striking absence of recent research conducted within European contexts. Staff often lack formal training, and there is a notable absence of post-burn sexual health educational resources, leaving many healthcare providers unprepared to initiate conversations about intimacy and sexual functioning. In many rehabilitation centers, no individual is designated as responsible for addressing these sensitive yet important issues.

Findings from staff surveys on sexuality and intimacy indicate consensus that patients should not bear the responsibility of initiating discussions on these topics [30,33]. Staff members acknowledge a lack of education in this area and admit that the topic is only sporadically raised by professionals. While psychologists are often viewed as the most appropriately equipped to address sexuality concerns, many clinicians report feeling comfortable discussing the topic themselves, provided that a more structured and supported approach is available.

Despite the recognized importance of addressing sexuality in rehabilitation settings, several barriers continue to impede healthcare professionals from engaging in these discussions. Staff frequently report feeling unprepared and even irresponsible when initiating conversations about sexuality [30,33]. The absence of clearly defined responsibilities within the multidisciplinary burn care team contributes to the tendency to defer these discussions to other professionals, further perpetuating the neglect of this domain [30]. As a result, sexuality remains largely unaddressed in many rehabilitation programs [30].

In addition, practical constraints—such as limited time and a lack of private, protected environments—often prevent meaningful dialog, with discussions sometimes taking place in shared rooms or during inpatient stays where confidentiality cannot be ensured. There is also a perception among staff that addressing sexuality may be of little value in settings where no specialized services or treatment options are available, thereby discouraging engagement with this aspect of care [30].

### 3.5. Practical Support Tools

Based on these recommendations, easily accessible brochures can help convey these key messages to both patients and healthcare professionals. Based on the existing literature, grey literature and expert opinion, two evidence-informed information tools were developed, one designed for healthcare professionals to increase confidence and competence in addressing these sensitive topics, and a second created for patients to provide reassurance, guidance, and validation throughout their recovery journey. These practical tools are intended to facilitate communication, reduce stigma, and promote a shared understanding of the challenges and possibilities related to sexuality and intimacy after burn injury:

1. A poster for healthcare professionals, designed to raise awareness and build confidence in initiating conversations about sexuality and intimacy in burn care, view Supplementary Materials S1;
2. A brochure for patients, designed to offer guidance, and support throughout their recovery journey, view Supplementary Materials S2.

Based on the foregoing, the following key points can be put in place by professionals to help support patients in these issues.

- Normalize and prioritize sexual well-being as part of routine burn rehabilitation.
- Screen proactively using risk factors and validated tools integrated with standard assessments.
- Professionals should: Provide permission to discuss concerns; offer Limited Information; Give Specific Suggestions when appropriate; Refer for Intensive Therapy if needed. (PLISSIT model gives a structured approach)
- Ensure privacy and dignity: schedule conversations in private rooms; use respectful, non-dismissive language.
- Embed sexual well-being goals in individualized care plans and review progress in MDT meetings. Long-term follow-up should include psychosocial and sexual health.
- Strengthen pathways for complex cases (urology, gynecology, sex therapy, counseling; trauma-related dysfunction).
- Clinicians need training and resources to address these topics competently. Burn teams should appoint team members who take the lead on documentation and implementation.
- Consistently respect and center patients’ experiences, perspectives, and preferences.

## 4. Discussion

Advances in burn care have contributed significantly to improving survival rates, albeit sexuality and intimacy remain key areas that have received limited attention. Despite the plethora of post-burn rehabilitation programs, aspects that target post-burn sexual well-being and intimacy remain either limited or non-existent. Issues regarding sexuality and intimacy remain shrouded in secrecy, even in the most liberal societies, and both burn survivors and burn care staff struggle to discuss these sensitive issues. These issues informed the current study to develop practice recommendations that can support burn care staff in addressing this often-neglected aspect of post-burn recovery.

This study identified several key factors significantly influencing sexual well-being and sexual satisfaction following burn injury. These include the TBSA, the depth of the burn, the specific location (notably the face or intimate areas), the duration of hospitalization, and the presence and severity of scarring. These physical and experiential factors profoundly shape a survivor’s perception of their altered body image and create significant challenges in adapting to their post-burn identity [16,34]. Consequently, they often lead to difficulties with intimacy and diminished sexual well-being [35]. Understanding this plethora of factors provides crucial insight for identifying which subgroups of burn survivors are potentially most vulnerable to experiencing these specific challenges. Furthermore, the sensitivity surrounding post-burn body changes and sexuality often leads both survivors and their partners to feel hesitant or embarrassed to initiate discussions about sexual health with healthcare providers. Therefore, recognizing the presence of these specific risk factors (e.g., extensive scarring, facial burns, prolonged hospitalization) can serve as valuable clinical indicators, prompting healthcare teams to proactively and sensitively initiate essential conversations about sexual well-being and support needs.

This study underscores persistent challenges within burn care related to addressing survivors’ sexual well-being. Crucially, it highlights a significant gap: many burn care staff report feeling inadequately trained and uncomfortable initiating discussions on this sensitive topic [30,31]. While various rehabilitation programs exist, they frequently neglect to incorporate essential components addressing sexual health and intimacy after burn injury [3,25]. Even when such support is theoretically available, profound socio-cultural factors, including stigma, taboos, communication barriers, and diverse value systems, often create substantial obstacles to effectively addressing these needs in practice [36]. Consequently, a fundamental shift towards a culturally sensitive, person-centered approach is imperative to navigate these complex challenges and provide holistic care. To bridge these critical gaps, sexual health and intimacy topics must be systematically embedded within the foundational training curricula for all burn care practitioners [25]. This foundational knowledge is essential to building confidence and competence, enabling staff to proactively and comfortably engage with survivors on these issues. Continuing professional development programs can also help to equip burn care staff with the competencies to identify and manage issues relating to post-burn sexual well-being. Practical recommendations highlighted in this study include that patient education material may help burn patients to feel comfortable raising sexual issues, rather than shy away from these. Also, we include an information poster for the burn care team and centers, which can guide burn care staff in initiating conversations on sexual well-being and supporting the patient through the process.

Recognizing the profound sensitivity inherent in discussions of sexual well-being after burn injury, creating a physically and psychologically safe environment is paramount for enabling survivors to engage openly [37]. The typical burn care setting, often characterized by high activity levels, limited space, and inherent challenges to confidentiality, can feel unsafe for disclosing such intimate concerns [38]. Therefore, ensuring strict privacy is not merely beneficial but a crucial prerequisite for effective assessment and support. Concrete steps must be taken to proactively allocate dedicated, private consultation rooms for these sensitive conversations. This involves scheduling discussions in advance within spaces specifically designed to minimize interruptions and auditory/visual exposure. Providing a controlled, confidential setting directly combats the environmental barriers present in busy units, actively fostering the trust and safety necessary for survivors to voice their needs regarding intimacy, body image, and sexual function as part of their holistic rehabilitation.

The majority of patients want clinicians to know more about them as a person [39]; some will wish to share information, and others will not. We should always try to respect and understand the diversity of patients’ personal experiences, perspectives and preferences.

Building upon the identified need for proactive intervention, integrating routine screening for sexual health concerns is a critical, yet often overlooked, component of comprehensive burn rehabilitation. While current rehabilitation programs typically screen for physical and psychosocial sequelae, the assessment of sexual well-being remains notably underdeveloped and insufficient. Although instruments like the Burn Specific Health Scale-Brief (BSHS-B) include some items related to sexuality [40], their limited scope (only 1–2 questions) fails to capture the nuanced spectrum of potential challenges—including distress with altered self, intimacy difficulties, and relationship impacts—that significantly affect survivors’ lives. Given the current scarcity of validated, burn-specific sexual well-being screening tools, utilizing well-established generic instruments, such as the Sexual Dysfunction Evaluation Tool (SEXDET) [41] or similar validated measures, represents a necessary and pragmatic interim solution. These sexual health screenings should be systematically incorporated into the standard assessment protocol alongside evaluations of physical function and psychological status. This routine integration ensures a truly holistic understanding of the survivor’s recovery journey, enabling clinicians to identify unmet needs early, initiate timely conversations, and tailor support effectively within the framework of person-centered care.

Table 1 gives an overview of different questionnaires and tools that could be useful for the evaluation of Intimacy, Sexuality and Body Image with the concept or outcome they evaluate and a short description of their content.

Given the general lack of practice recommendations to address post-burn sexual well-being issues, the infographics developed as part of this study offer a practical guide. This includes the strategies that can be employed to facilitate sexual well-being and intimacy discussions, long-term support and follow-up, and empower patients and their significant others. While the effectiveness of the infographics is beyond the scope of the current study, the comprehensive nature of the practical guide provided holds the promise of facilitating discussions around post-burn sexual well-being and intimacy. Future studies may consider employing these infographic resources as part of aftercare interventions to evaluate their impact.

## 5. Conclusions

The literature review clearly highlighted a gap between the significant impact burns have on survivors’ sexuality and intimacy, and therefore the need to address these issues, and the reality of an unease in discussing these issues, both on the part of patients and caregivers.

This persists despite the availability of known risk factors that could trigger proactive screening and the recognized need for safe, respectful environments in which to discuss these issues. Routine use of sexual well-being assessments also remains limited, particularly given the lack of burn-specific validated tools.

To begin the process of gradually integrating the issue of sexuality into burn care, it was deemed appropriate to convene an international, multidisciplinary group of experts to develop an information resource for burn survivors and a set of recommendations for healthcare professionals.

The existence of such resources would immediately highlight the importance of this subject, enabling teams to create protocols and contexts for addressing it and supporting patients with their sexuality and intimacy. These resources help signal the importance of sexual well-being, encourage teams to create appropriate protocols and spaces for discussion, and reassure patients that their concerns are acknowledged rather than minimized. For survivors, the brochure offers practical guidance and serves as an accessible first point of contact, supporting and normalizing help-seeking.

Finally, embedding sexuality and intimacy within burn care requires ongoing professional training and the identification of dedicated resource persons within each team. These individuals can champion the topic, ensure consistent practices, and provide support for both colleagues and patients.

## Figures and Tables

**Table 1:** Overview of questionnaires related to Intimacy, Sexuality and Body Image with outcome and description.

Outcome	Questionnaire	Description	Reference/Author
Quality of Life	BSHS-B	Burn specific quality of life questionnaire with 9 domains of which Sexuality and Body image	Kildal et al. [40]
Sexual functioning	QSF	A generic 40-item questionnaire with psycho-somatic quality of life, sexual activity, sexual (dys)function—self-reflection and sexual (dys)function—partner’s view	Heinemann et al. [42]
Sexual functioning	SEXDET	A generic visual digital tool with a pie chart based on the biopsychosocial-sexual-relationship model for conversations with clinician on possible causes of the sexual dysfunction (relationship-related, environmental, biological, psychological and sexual)	Burté et al. [41]
Body Image	SWAP	burn-specific measure of body image with subjective appraisal and social–behavioral components of body image	Lawrence. [43]
Body Image	BES	generic measure of body image with 3 subscales: (1) general feelings about appearance, (2) weight satisfaction and (3) evaluations of how others see their body or appearance	Mendelson et al. [44]
Sexual Satisfaction	NSSS-S	Generic measure on sexual satisfaction two subscales: with Ego-Centered Subscale (items 1–10) and the partner- and Activity-Centered Subscale (items 11–20) 20 items with 5 point Likert scale (1–5);	Štulhofer [45]
Marital Satisfaction	MMQ	Generic tool on marital satisfaction 20 items, 9 point Likert scale (0–8);	Arrindell et al. [46]
Social Participation	LIBRE	Burn-specific questionnaire on social participation 126 items with 6 subscales; with 15 items on Sexual relationships and 28 items on Romantic relationships	Kazis et al. [47]
Sexual Well-Being	CSWBIS	A scar-specific questionnaire with 5 items, a 4-point Likert scale (1–4); impact on sexual well-being and career due to the scar	Garg et al. [48]
Aftercare Problem List	APL	43 items divided with 9 domains: scars, daily life functioning, scars treatment, body perceptions, stigmatization, intimacy, mental health, relationships, financial concerns, positive coping + a distress thermometer and preference to discuss results with healthcare provider	Van Loey et al. [49]

BSHS-B = Burn Specific Health Scale-Brief, QSF = Quality of Sexual Function, SEXDET = Sexual Dysfunction Evaluation Tool, SWAP = Satisfaction with Appearance Scale, BES = Body Esteem Scale for Adolescents and Adults, NSSS-S = New Sexual Satisfaction Scale Short Form, MMQ = Maudsley Marital Questionnaire, LIBRE = Life Impact Burn Recovery Evaluation Profile, CSWBIS = Career and Sexual Well-Being Impact of Scars, Aftercare Problem List (APL).

## Data Availability

No new data were created or analyzed in this study. Data sharing is not applicable to this article.

## Supplementary Materials

The following supporting information can be downloaded at: https://www.mdpi.com/article/10.3390/ebj7020026/s1, Supplementary Materials S1: Information folder for patients; Supplementary Materials S2: Information poster for Health Care Clinicians.

## References

[1] Connell K.M., Coates R., Wood F.M. (2015). Burn injuries lead to behavioral changes that impact engagement in sexual and social activities in females. Sex. Disabil..

[2] Cheng W., Shen C., Zhao D., Zhang H., Tu J., Yuan Z., Song G., Liu M., Li D., Shang Y. (2019). The epidemiology and prognosis of patients with massive burns: A multicenter study of 2483 cases. Burns.

[3] Bayuo J., Wong F.K.Y. (2021). Issues and concerns of family members of burn patients: A scoping review. Burns.

[4] Dyer K., Das Nair R. (2013). Why don’t healthcare professionals talk about sex? A systematic review of recent qualitative studies conducted in the United Kingdom. J. Sex. Med..

[5] Kool M.B., Geenen R., Egberts M.R., Wanders H., Van Loey N.E. (2017). Patients’ perspectives on quality of life after burn. Burns.

[6] Whoqol Group (1995). The World Health Organization quality of life assessment (WHOQOL): Position paper from the World Health Organization. Soc. Sci. Med..

[7] Esmail S., Esmail Y., Munro B. (2001). The Role of Health Care Professionals in Providing Options and Alternatives for Couples. Sex. Disabil..

[8] Fobair P., Stewart S.L., Chang S., D′ONofrio C., Banks P.J., Bloom J.R. (2006). Body image and sexual problems in young women with breast cancer. Psychooncology.

[9] Bayuo J., Wong A.K.C., Wong F.K.Y., Baffour P.K., Kuug A.K. (2024). Sexual role functioning, sexual satisfaction, and intimacy after surviving burn injuries: A scoping review of associated factors, screening tools, and burn care staff preparedness. J. Burn Care Res..

[10] Roy M., Payette H. (2012). The body image construct among Western seniors: A systematic review of the literature. Arch. Gerontol. Geriatr..

[11] Burychka D., Miragall M., Baños R.M. (2021). Towards a comprehensive understanding of body image: Integrating positive body image, embodiment and self-compassion. Psychol. Belg..

[12] Connell K.M., Coates R., Wood F.M. (2013). Sexuality following burn injuries: A preliminary study. J. Burn Care Res..

[13] Huang Y.K., Su Y.J. (2021). Burn severity and long-term psychosocial adjustment after burn injury: The mediating role of body image dissatisfaction. Burns.

[14] Cleary M., Kornhaber R., Thapa D.K., West S., Visentin D. (2020). A quantitative systematic review assessing the impact of burn injuries on body image. Body Image.

[15] Bakker A., Maertens K.J., Van Son M.J., Van Loey N.E. (2013). Psychological consequences of pediatric burns from a child and family perspective: A review of the empirical literature. Clin. Psychol. Rev..

[16] Ohrtman E.A., Shapiro G.D., Wolfe A.E., Trinh N.-H.T., Ni P., Acton A., Slavin M.D., Ryan C.M., Kazis L.E., Schneider J.C. (2020). Sexual activity and romantic relationships after burn injury: A Life Impact Burn Recovery Evaluation (LIBRE) study. Burns.

[17] Rimmer R., Rutter C. Understanding Intimacy and Sexuality After Burn Injury. https://phoenix-society.org/resources/understanding-intimacy-and-sexuality-after-burn-injury.

[18] Rimmer R.B., Rutter C.E., Lessard C.R., Pressman M.S., Jost J.C., Bosch J., Foster K.N., Caruso D.M. (2010). Burn care professionals’ attitudes and practices regarding discussions of sexuality and intimacy with adult burn survivors. J. Burn Care Res..

[19] Pandya A.A., Corkill H.A., Goutos I. (2015). Sexual function following burn injuries: Literature review. J. Burn Care Res..

[20] Hordern A.J., Currow D.C. (2003). A patient-centred approach to sexuality in the face of life-limiting illness. Med. J. Aust..

[21] Öster C., Sveen J. (2015). Is sexuality a problem? A follow-up of patients with severe burns 6 months to 7 years after injury. Burns.

[22] Dejours C. (2009). Le corps, comme «exigence de travail» pour la pensée. Psychopathologie des Limites.

[23] Deng H., Genovese T.J., Schneider J.C. (2023). A narrative review of outcomes in burn rehabilitation based on the International Classification of Functioning, Disability, and Health. Phys. Med. Rehabil. Clin..

[24] Akdeniz F., Şekerci Ç.A., Tanıdır Y., Yılmaz Y., Çam K. (2022). Erectile dysfunction in patients with major burn injury: The significance of follow-up. Turk. J. Trauma Emerg. Surg..

[25] Hurley A., King I.C., Perry F.M., Dheansa B.S. (2022). Addressing sexual function in adult burns victims: A multidisciplinary survey of current practice in UK burn units. Burns.

[26] Levi B., Kraft C.T., Shapiro G.D., Trinh N.-H.T., Dore E.C., Jeng J., Lee A.F., Acton A., Marino M., Jette A. (2018). The Associations of Gender with Social Participation of Burn Survivors: A Life Impact Burn Recovery Evaluation Profile Study. J. Burn Care Res..

[27] Abel N.J., Klaassen Z., Mansour E.H., Marano M.A., Petrone S.J., Houng A.P., Chamberlain R.S. (2012). Clinical outcome analysis of male and female genital burn injuries: A 15-year experience at a Level-1 Burn Center. Int. J. Urol..

[28] Cato L.D., Shepler L.J., McMullen K., Roaten K., Kazis L.E., Ryan C.M., Schneider J.C. (2023). T3 Sexual Satisfaction and Association with Psychosocial Outcomes Among Burn Survivors. J. Burn Care Res..

[29] Gonçalves N., Melo A.d.S., Caltran M.P., Pedro I.C.d.S., Pan R., Nascimento L.C., Rossi L.A. (2014). Sexuality in burn victims: An integrative literature review. Burns.

[30] Piccolo M.S., Daher R.P., Gragnani A., Ferreira L.M. (2011). Sexuality after burn in Brazil: Survey of burn health-care workers. Burns.

[31] Ahmad I., Masoodi Z., Akhter S., Khurram F. (2013). Aspects of Sexual Life in Patients After Burn. J. Burn Care Res..

[32] Kazemzadeh J., Rabiepoor S., Alizadeh S. (2022). Satisfaction with appearance and sexual satisfaction in women with severe burn injuries. Int. J. Impot. Res..

[33] Piccolo M.S., Gragnani A., Daher R.P., Scanavino M.d.T., de Brito M.J., Ferreira L.M. (2013). Burn Sexuality Questionnaire: Brazilian translation, validation and cultural adaptation. Burns.

[34] Van Loey N.E. (2020). Psychological impact of living with scars following burn injury. Textbook on Scar Management: State of the Art Management and Emerging Technologies.

[35] Simons M., Price N., Kimble R., Tyack Z. (2016). Patient experiences of burn scars in adults and children and development of a health-related quality of life conceptual model: A qualitative study. Burns.

[36] Bayuo J., Wong F.K.Y., Wong A.K.C., Baffour P.K., Chung L.Y.F. (2024). A comprehensive nurse-led aftercare programme addressing post-burn sexual well-being of adult burn survivors: A randomised controlled trial. BMC Nurs..

[37] Fennell R., Grant B. (2019). Discussing sexuality in health care: A systematic review. J. Clin. Nurs..

[38] Rafii F., Oskouie F., Nikravesh M. (2007). Caring behaviors of burn nurses and the related factors. Burns.

[39] Zimmerman D.L., Min D.J., Davis-Collins A., DeBlieux P. (2020). Treating patients as people: What do hospital patients want clinicians to know about them as a person?. J. Patient Exp..

[40] Kildal M., Andersson G., Fugl-Meyer A.R., Lannerstam K., Gerdin B. (2001). Development of a brief version of the Burn Specific Health Scale (BSHS-B). J. Trauma Acute Care Surg..

[41] Burté C., Bei S., Becquet E., Huyghe E., Maziashvili T. (2025). Development of a new digital sexual health assessment tool to help patients better understand the factors involved in their sexual disorders–“The Sexual Dysfunction Evaluation Tool”(SEXDET). Fr. J. Urol..

[42] Heinemann L.A., Potthoff P., Heinemann K., Pauls A., Ahlers C.J., Saad F. (2005). Scale for Quality of Sexual Function (QSF) as an outcome measure for both genders?. J. Sex. Med..

[43] Lawrence J.W., Heinberg L.K., Roca R., Munster A., Spence R., Fauerbach J. (1998). Development and validation of the Satisfaction with Appearance Scale: Assessing body image among burn-injured patients. Psychol. Assess..

[44] Mendelson B.K., Mendelson M.J., White B.R. (2001). Body Esteem Scale for adolescents and adults. J. Pers. Assess..

[45] Štulhofer A., Buško V., Brouillard P. (2010). Development and bicultural validation of the new sexual satisfaction scale. J. Sex Res..

[46] Arrindell W.A., Schaap C.A.S. (1985). The Maudsley Marital Questionnaire (MMQ): An extension of its construct validity. Br. J. Psychiatry.

[47] Kazis L.E., Marino M., Ni P., Bori M.S., Amaya F., Dore E., Ryan C.M., Schneider J.C., Shie V., Acton A. (2017). Development of the life impact burn recovery evaluation (LIBRE) profile: Assessing burn survivors’ social participation. Qual. Life Res..

[48] Garg S.P., Weissman J.P., Chwa E.S., Galiano R.D. (2023). Content Validity of a Novel Scar Assessment Tool Evaluating the Career and Sexual Well-being Impact of Scars. Plast. Reconstr. Surg. Glob. Open.

[49] Van Loey N.E.E., Boersma-van Dam E., Boekelaar A., van de Steenoven A., de Jong A.E.E., Hofland H.W.C. (2024). Development and Testing of the Aftercare Problem List, a Burn Aftercare Screening Instrument. Eur. Burn J..

