# Supplementary material for: Recommendations on Sexuality and Intimacy After Burn Injuries
Source: Eur Burn J. 2026 May 12;7(2):26. doi: 10.3390/ebj7020026 (PMC13214789; doi:10.3390/ebj7020026)
Supplement: Supplementary file 1 [file ebj-07-00026-s001.zip › Supplementary Materials S1 Information folder for patients 10032026.pdf]

Practical Tips for Daily Living

Do things or take important steps together with someone else: Everything is easier with someone you trust, especially on days when you may not feel particularly well. If you don't have someone available (family or friends), don't hesitate to reach out to support groups, or professionals who could accompany or assist you.
Your body can adapt: Different positions or forms of intimacy will help you to share intimacy despite your movement limitations or sensory changes.
If you have a partner, try to think about her/his/their place: This experience might have focused her/him/them in a caregiving role and/or may have distanced her/him/them from you. Intimacy can be a way to recreate simple links.

Seeking Professional Help

Choose your trusted professional: Choose someone you feel comfortable with, to talk about these topics.
Psychological Support: Counseling can help address emotional and psychological challenges.
Medical Follow-Up: Regular check-ups to monitor physical recovery and address any complications. Talk to your doctor if you have lower energy or decreased
desire.
Sexual therapy: Ask for a qualified specialist support in case of need.

Staying Positive and Motivated

Set Realistic Goals: Focus on small, achievable steps.
Celebrate Progress: Acknowledge and celebrate even small improvements.
Stay Connected: Maintain relationships with friends and or family for emotional support.

Helpful Resources Center specific and contacts about sexuality and Intimacy
--- Editable Section for Local Customization ---

Please add your center-specific resources below:
National and/or Local Burn Survivor Association(s): [Add name, website, email, phone number]
Clinical Psychologist: [Add name, website, email, phone number]
Sexual Health Center and or After-care Center: [Add name, website, email, phone number]

Authors: Stefania Simone, Sabrina Belemkasser, Jonathan Bayuo, Jill Meirte
Feel free to contact the authors if you have questions, remarks or suggestions.
Correspondence: jill.meirte@uantwerpen.be

www.euroburn.org
Information Brochure for Burn Survivors

Intimacy and sexuality post-burn

Understanding Burns and Recovery

Body Image and Self-Esteem

Intimacy and Sexuality

Practical Tips for Daily Living

Seeking Professional Help

Staying Positive and Motivated

Your burn injury might have changed a lot of things and recovering may require immense effort and resilience or strength and persistence. In your rehabilitation, you might find yourself concerned with more than just your physical recovery. Feelings about your body and your relationships with others, including intimacy and sexuality, are common concerns after a burn injury.

Feeling good about your body and having positive feelings towards intimacy and sexuality may be vital aspects of your overall well-being. You have the right to have these concerns addressed. We understand that discussing these issues can be sensitive and challenging, but remember that sexuality is a natural part of life. Our goal is to support you in every aspect of your recovery, helping you move towards a fulfilling and complete healing journey. Everyone is different and some things may take more or less time and or will become more or less important throughout time.

This brochure is primarily designed to support you as the patient, but we also want to acknowledge that partners who are going through this experience alongside you may face their own challenges. Partners who are part of this journey may also experience challenges, and we encourage them to seek help or talk about their feelings if they wish.

Understanding Burns and Recovery

Physical Changes: Common problems include scarring, changes in skin texture, physical limitations and hormonal imbalance (including fertility issues), which can lead to low energy, sleep patterns, limited physical function, skin sensitivity, and altered appearance.

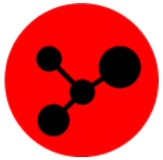

Psychological Impact: Feelings of shame, insecurity, and altered body image are common. These can affect self-esteem and social interactions.

Role change effects: Changes in roles and relationships after a burn injury may lead to feelings of being less needed, not having control over one's life, or having difficulty fulfilling other roles, such as being a parent or partner.

Importance of Rehabilitation: Engaging in physical and psychological rehabilitation can significantly improve quality of life.

Body Image and Self-Esteem

Body Image: The mental picture one has of their body, which can be affected by burn injuries.

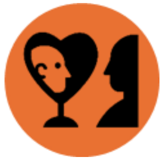

Improving Body Image:

Positive Self-Talk: Focus on strengths and progress.

Manage your scar as you see fit: Some feel the need to hide their scars and some don't. If at any time they cause discomfort or affect how you feel, there are gentle options like makeup or decorative coverings (such as wigs).

Support Groups: Connect with others who have similar experiences.

Sports / Exercise: Sport and or exercise can give you a great sense of body and self-esteem. It strengthens the body, you see and feel personal progress and it can be a great mental support, which in turn contributes to positive self-esteem.

Good environment: Your family and friends may be very important resources in dealing with the consequences of the burn injury.

Intimacy and Sexuality

Impact on Relationships: Burns can affect intimate relationships due to physical changes and emotional stress. Changes can be less interest in intimacy and sex, lower (sexual) arousal, less feeling or increased sensitivity, menstrual period breaks,…

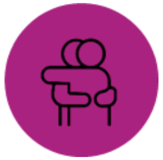

Communication is Key: Open, honest communication about needs and concerns, whether with a partner, close friend, or trusted professional. It is important to know that it may take some time of trial and error to find what works.

Explore New Forms of Intimacy: Focus on emotional closeness and non-sexual forms of intimacy. Sometimes it can be enough just to feel loved and safe, without feeling the need to perform. Over time, you will become more confident and feel better about yourself and your partner.

Practical Tips for Daily Living

Focus on your own needs, wishes, and desires

Moisturize Regularly: After a burn injury, newly healed skin may be fragile and prone to shearing. Keep the skin moisturized to reduce friction, blistering or tearing of the skin and to reduce itching.

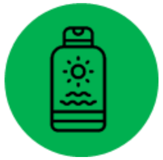

Pain Management: Take pain medication as prescribed, especially before engaging in activities.

Plan Activities: Schedule activities for times when energy levels are highest.

Exercise and activity: Try to incorporate exercises into your daily routine, e.g. in the morning after getting up, preferably do exercises that are fun or enjoyable. No big program, keep it as simple as possible so that you stay motivated.
