# Supplementary material for: Recommendations on Sexuality and Intimacy After Burn Injuries
Source: Eur Burn J. 2026 May 12;7(2):26. doi: 10.3390/ebj7020026 (PMC13214789; doi:10.3390/ebj7020026)
Supplement: Supplementary file 1 [file ebj-07-00026-s001.zip › Supplementary Materials S2 Information poster for Health Care Clinicians 10032026.pdf]

Intimacy and sexuality post-burn

Burn injuries can affect much more than the body, they can influence how people feel about closeness, touch, privacy, and sexuality. For some patients, intimacy may feel distant or even disappear for a while. As healthcare professionals, it's important to remember that patients are not their injuries. During dressing changes, bathing, or assessments, be mindful to protect their privacy, cover body parts whenever possible, and respect boundaries. Small actions can make a big difference in helping someone feel safe, dignified, and seen as a person. While wounds and scars may appear to be healing well from a clinical perspective, the emotional experience can be very different. Avoid comments that might feel dismissive or patronising, such as "This looks great!" or "You're doing so well," without first checking how the patient feels about their own healing. Always speak and act in ways that honour autonomy and maturity. Patients, including adolescents and young adults, deserve to be engaged as capable partners in their care. Intimacy and sexuality are meaningful parts of life. It's okay for patients to have questions or concerns, and it's important for us to create space where these conversations can happen safely and respectfully.

Addressing Body Image and Sexuality

- **Body Image:** What is the impact of burn injury on a patient's body image/self-image.
- **Sexuality and Intimacy:** Recognize that these are crucial components of overall well-being.
- **Create a Safe Space:** Allow open conversations about intimacy, sexual health and body image. Who could the patient turn to in the multi-disciplinary team? Who will talk about these themes with patients and carers? Include these topics in a standard assessment list.

Communication Strategies

- **P-LI-SS-IT Model: see other side for a concrete example**
    - **P**ermission: Give patients permission to discuss intimacy and sexuality.
    - **L**imited **I**nformation: Provide basic information about sexual health.
    - **S**pecific **S**uggestions: Offer practical advice tailored to the patient's situation.
    - **I**ntensive **T**herapy: Refer to specialists (e.g., sexologist, counselor, therapist) when necessary.
- Use **Who, How and When**: Use Open questions "How is intimacy since you burn injury?"

Training and Education for Clinicians

- **Ongoing Training:** Follow workshops, webinars,… on post-burn care, focusing on psychological and sexual health. If possible (expertise in own center) organize interactive education addressing this in team.
- **Resource Availability:** Ensure access to up-to-date resources, recommendations and guidelines.

Long-Term Support and Follow-Up

- **Outpatient Follow-Up:** Organize regular psychosocial follow-ups for patients
- **Peer support/survivor groups:** Point patients to possible peer support (e.g. burn camp, existing peer support groups,…). If possible organize a round table discussion within peer/survivor support group on the topic of Intimacy sexuality.
- **Tailored Care Plans:** Develop individualized care plans addressing long-term needs.

Empowering our Patients

- **Self-Care:** Teach patients about skin and scar (self) care, pain management, and other techniques or strategies that increase self-confidence and control.
- **Support Networks:** Encourage patients if interested to join burn survivor networks and support groups and provide references and contact information of such networks.
- **Positive Reinforcement:** Reinforce progress and encourage a proactive approach to recovery.

- **Reclaiming Their Body Image**: Support patients in the process of seeing themselves again. Offer the possibility to use a full mirror during dressing changes or showering, and always allow mirror use when a patient requests it. This helps restore autonomy and fosters a sense of ownership over their body during recovery.

Comprehensive Burn Care

- **Holistic Approach:** Address physical, psychological, and social aspects of burn recovery.
- **Interdisciplinary Team:** Collaborate with surgeons, psychologists, sexologists, physical and occupational therapists, social workers, etc. Work interdisciplinary or transdisciplinary to create a joint patient centered care plan. Plan interdisciplinary meetings for care planning.

Practical Tips for Clinical Practice

- **Assessments and follow-up:** try to check for these aspects in your assessments and follow-up and use if possible use tools like the Burn Specific Health Scale (BSHS), Body Image Scale (BIS), After-care checklist to objectify these aspects over time.
- **Patient Education:** Provide educational materials on managing scars, pain, and emotional well-being.
- **Partner and Family Involvement:** Encourage relatives or partners to participate in the care process.

Helpful Resources Center specific and contacts about sexuality and Intimacy

*--- **Editable Section for Local Customization** ---*

Please add your center-specific resources below:

*• **National and or Local Burn Survivor Association(s): [Add name, website, email, phone number]***

*• Clinical Psychologist: **[Add name, website, email, phone number]***

*• Sexologist: **[Add name, website, email, phone number]***

*• Sexual Health Center and/or After-care Center: **[Add name, website, email, phone number]***

Questions to discuss or reflect on in the multidisciplinary team (meetings)

• Who in the team will be taking the lead in your centre for the assessments, documentation for patients,…

• How do you assure implementation in your centre and or unit…

• Are there trainings, resources and persons in place if colleagues have questions about these topics

Authors: Stefania Simone, Sabrina Belemkasser, Jonathan Bayuo, Jill Meirte
Feel free to contact the authors if you have questions, remarks or suggestions.
Correspondence: jill.meirte@uantwerpen.be
